# CRISPR MultiTargeter: A Web Tool to Find Common and Unique CRISPR Single Guide RNA Targets in a Set of Similar Sequences

**DOI:** 10.1371/journal.pone.0119372

**Published:** 2015-03-05

**Authors:** Sergey V. Prykhozhij, Vinothkumar Rajan, Daniel Gaston, Jason N. Berman

**Affiliations:** 1 Department of Pediatrics, Dalhousie University, Halifax, Nova Scotia, Canada; 2 Department of Microbiology and Immunology, Dalhousie University, Halifax, Nova Scotia, Canada; 3 Department of Pathology, Dalhousie University, Halifax, Nova Scotia, Canada; Osaka University, JAPAN

## Abstract

Genome engineering has been revolutionized by the discovery of clustered regularly interspaced palindromic repeats (CRISPR) and CRISPR-associated system genes (Cas) in bacteria. The type IIB *Streptococcus pyogenes* CRISPR/Cas9 system functions in many species and additional types of CRISPR/Cas systems are under development. In the type II system, expression of CRISPR single guide RNA (sgRNA) targeting a defined sequence and Cas9 generates a sequence-specific nuclease inducing small deletions or insertions. Moreover, knock-in of large DNA inserts has been shown at the sites targeted by sgRNAs and Cas9. Several tools are available for designing sgRNAs that target unique locations in the genome. However, the ability to find sgRNA targets common to several similar sequences or, by contrast, unique to each of these sequences, would also be advantageous. To provide such a tool for several types of CRISPR/Cas system and many species, we developed the CRISPR MultiTargeter software. Similar DNA sequences in question are duplicated genes and sets of exons of different transcripts of a gene. Thus, we implemented a basic sgRNA target search of input sequences for single-sgRNA and two-sgRNA/Cas9 nickase targeting, as well as common and unique sgRNA target searches in 1) a set of input sequences; 2) a set of similar genes or transcripts; or 3) transcripts a single gene. We demonstrate potential uses of the program by identifying unique isoform-specific sgRNA sites in 71% of zebrafish alternative transcripts and common sgRNA target sites in approximately 40% of zebrafish duplicated gene pairs. The design of unique targets in alternative exons is helpful because it will facilitate functional genomic studies of transcript isoforms. Similarly, its application to duplicated genes may simplify multi-gene mutational targeting experiments. Overall, this program provides a unique interface that will enhance use of CRISPR/Cas technology.

## Introduction

In the last two decades bacterial and archeal species have been recognized to possess adaptive immunity against molecular pathogens such as viruses [1]. This immunity is based on clustered regularly interspaced palindromic repeats (CRISPR) and spacers homologous to the targeted pathogens as well as Cas (CRISPR associated sequence) proteins. Spacer sequences originate from protospacer regions, which are also characterized by specific protospacer adjacent motifs (PAM) necessary for their cleavage and insertion into the CRISPR fragment [2,3]. CRISPRs and corresponding spacers are transcribed and processed into non-coding crRNAs, which in a complex with specific Cas proteins can cleave DNA recognized by the spacer RNA region. CRISPR/Cas systems are diverse and are classified into three types based on the sequence, the location of the PAM, and specific Cas genes [4]. Of all the known CRISPR/Cas systems, the type IIB system was adopted for research use when it was shown that a single guide RNA (sgRNA) generated by the joining of crRNA and tracrRNA from *Streptococcus pyogenes* can successfully program Cas9 to cleave different DNA sequences containing an NGG PAM sequence after the spacer sequence in the same strand [5]. Such artificial sgRNAs, together with Cas9, were first applied to human cells [6,7] and then to an increasing number of other species (reviewed in [8]). Moreover, variant CRISPR/Cas9 systems (*e*.*g*. *Neisseria meningitides* Cas9 (NmCas9) cutting at NNNNGATT PAM [9] or Streptococcus thermophilus Cas9 (StCas9) cutting at NNAGAAW PAM [10]) have also been engineered for experimental use and may find novel applications [11].

Basic computational research on the CRISPR/Cas systems focused on identifying CRISPR sites in bacterial and archeal genomes, which led to the development of such tools as CRISPR finder and CRISPRdb [12]. Adoption of CRISPR/Cas for experimental work also stimulated development of software programs for generating sgRNAs and finding their target sites (Table 1). ZiFiT (http://zifit.partners.org/ZiFiT/) [13] is one of the earliest available programs for quick searches of potential Cas9 sgRNA target sites in DNA sequences. Recent progress in sgRNA design software includes the implementation of off-target site search functionality to minimize the potential problems posed by off-target activities of sgRNA/Cas complexes, design of sgRNAs for the novel types of CRISPR/Cas systems, and new applications of existing enzymes. Optimized CRISPR Design (http://crispr.mit.edu/) by the Zhang Lab has enabled extensive off-target site analysis, but currently it is limited exclusively to “NGG” PAM and a sequence length of 250 nucleotides, with each run taking several minutes. Similarly, searching for sgRNA target sites with the CRISPR Direct tool (http://crispr.dbcls.jp/) from the Database Center for Life Sciences results in the output of a table of candidate sites with their sequences, main sequence features of those sites, as well as the number of unique matches in the genome and”12-mer + PAM” match numbers [14]. Cas9 Online Designer (http://cas9.wicp.net/) developed by Dayong Guo and a standalone software package sgRNAcas9 [15] are two additional programs that can check off-target sites directly during target site searches. Of all the currently available software for sgRNA design, the CHOPCHOP website (https://chopchop.rc.fas.harvard.edu/) stands out because of its speed, versatility, user-friendliness, dynamic graphical interface, and off-target prediction, but the species coverage is somewhat limited [16]. CRISPR/Cas9 targeting for different protein tagging experiments is supported by the E-CRISP website (http://www.e-crisp.org/E-CRISP/) [17], which also provides similar functionality to other programs. Interestingly, while this manuscript was in preparation, the CRISPRSeek Bioconductor package was revealed, implementing analysis of sgRNA targets in two sequences, somewhat similar to the workflows in the current software [18], but not as fully developed as in our software and also requiring R and Bioconductor skills for use. The targeting efficiency potential of sgRNAs is another very important topic beginning to be addressed by sgRNA design tools, the first of which is sgRNA Designer from the Broad Institute (http://www.broadinstitute.org/rnai/public/analysis-tools/sgrna-design) [19]. These authors prepared large pooled libraries of sgRNA vectors for a number of genes, quantified their targeting efficiency in a quantitative manner and developed a statistical model to predict the targeting efficiency score of sgRNAs based on its sequence. Focusing on many species from a specific phylogenetic group is another direction of CRISPR/Cas9 sgRNA design software as exemplified by flyCRISPR Optimal Target Finder (http://tools.flycrispr.molbio.wisc.edu/targetFinder/) [20]. Off-target prediction has also been implemented for already designed sgRNAs by groups who published CasOT [21], Cas-OFFinder (http://www.rgenome.net/cas-offinder/) [22] and GT-Scan (http://gt-scan.braembl.org.au/gt-scan/) [23]. CasOT and Cas-OFFinder are focused exclusively on CRISPR/Cas target sites and are somewhat restrictive in their parameters, while GT-Scan can accommodate a very broad target definition, is fast and user-friendly. Therefore, GT-Scan is the software of choice for verifying target sites identified using our software.

**Table 1:** Comparison of different sgRNA design software programs.

Tool	Type of CRISPR/Cas system	Sequence input	Support for Cas9 nickase	Comparison of multiple sequences	Off-target analysis	Scoring	Species support	Batch mode	Software type	Citation
**ZiFiT**	Type II only	Sequence only	**Yes**	No	No	No	N.A.	No	web	[13]
OptimizedCRISPR Design	Type II only	Sequence only	**Yes**	No	**Yes**	Off-target scoring	15	**Yes**	web	
CRISPR Direct	Type II Only	Sequence/Identifiers	No	No	**Yes**	Off-target scoring	18	No	web	[14]
Cas9 OnlineDesigner	Type II only	Sequenceonly	**Yes**	No	**Yes**	No	20	No	web	
CHOPCHOP	Different Type II	Sequence/Identifiers	No	No	**Yes**	Off-target scoring	19	No	web	[16]
E-CRISP	Different Type II	Sequence/Identifiers	**Yes**	No	**Yes**	Off-target scoring	21	No	web	[17]
sgRNAcas9	Type II only	Sequence only	**Yes**	No	**Yes**	Off-target scoring	N.A.	**Yes**	local	[15]
sgRNA Designer	Type IIonly	Sequence/Identifiers	No	No	No	**ActivityScore—type II**	N.A.	**Yes**	Web/local	[19]
CRISPRseek	Different Type II	Sequence only	**Yes**	**Yes**	**Yes**	Off-target scoring	N.A.	**Yes**	Bioc*	[18]
CRISPR MultiTargeter	Multiple types	Sequence/Identifiers	**Yes**	**Yes**	No	**ActivityScore—type II**	12	**Yes**	web	This Paper

*Bioc—Bioconductor package of the R programming and statistical environment

Despite this rapidly growing list of online resources, current software development for CRISPR/Cas systems has extensively focused on programs predicting target sites unique in the whole genome. We reasoned, however, that when there are two or more related sequence entities in the genome or the corresponding transcriptome, it would be useful to predict common sgRNA target sites present in ALL of these sequences, as well as unique target sites present only in ONE of the sequences. With this in mind, we developed CRISPR MultiTargeter, which is uniquely designed to work with duplicated genes and constitutive as well as alternative exons present in particular transcripts (compared to existing programs in Table 1). Such predicted target sites can be further tested using off-target site prediction software described above. CRISPR MultiTargeter can be applied to genomes of multiple species, arbitrary DNA sequences and supports different sgRNA target site specificities with their associated parameters. We also implemented the new scoring system for type II sgRNAs developed by Doench and colleagues [19]. Since our primary model system is the zebrafish (*Danio rerio*), a popular model system for understanding developmental, cellular and biochemical processes and mechanisms as well as for disease modeling, we performed genome-wide analyses of CRISPR MultiTargeter applications on transcript isoforms and duplicated genes as proof-of-concept. We propose that this software will simplify multiplex gene targeting and the mutational analysis of different transcript isoforms.

## Methods

### Database creation and sequence retrieval

When a user enters sequence identifiers, the program retrieves the sequences corresponding to those identifiers along with additional sequence information as prescribed by the algorithm. Refseq nucleotide sequences are retrieved from the Entrez system of National Center for Biotechnology Information and the retrieval process therefore places no restrictions on their species of origin. Storage and access to other sequence information has been implemented using SQLite3 database management system, which can be easily manipulated by SQL statements inside the Python scripts. We used Ensembl BioMart database as the source of gene, transcript and exon sequence information used in this software. The Biomart data were from Ensembl Genes 76 database version and the most recent genome assemblies for each species (*Homo sapiens*—GRCh38; *Mus musculus*—GRCm38.p2; *Rattus norvegicus*—Rnor_5.0; *Gallus gallus*—Galgal4; *Xenopus tropicalis*—JGI4.2; *Danio rerio*—Zv9; *Oryzias latipes*—HdrR; *Drosophila melanogaster*—BDGP5; *Caenorhabditis elegans*—WBcel235; *Arabidopsis thaliana*—TAIR10; *Oryza sativa japonica*—IRGSP-1.0; *Zea mays*—AGPv3). Our database has the following tables and fields: Genes (geneid, symbol, sequence, species), Exons (exonID, geneid, sequence, strand, chrstart, chrend, genestart, geneend) and Transcripts (transcriptID, geneid, sequence). Most of the sequence data contained in tables was inserted unaltered from original sources. The gene sequences were generated by sequential merging of all exons of each gene according to their coordinates. This feature of gene sequences enables exhaustive comparison of related genes, which is not possible when one compares transcript sequences that do not include some of the exons. To avoid erroneous target identification, candidate target sites are checked against the individual exon sequences.

### Web interface

The web interface of CRISPR MultiTargeter contains a front page with an explanation of the overall use of the program, graphical explanations, and the links to input pages for specific types of analyses, which will be described later. Irrespective of the analysis type, input web pages require users to input two types of information: the details of the sgRNA target site definition and input sequences, which will be used to find target sites. To define how sgRNA target sites are going to be searched, the user needs to specify the 5’ dinucleotide by choosing from three options (“NN”, “GN”, “GG”), the length of the target, and from which side the PAM sequence is located for that particular CRISPR/Cas system. The user can either choose the default “NGG” PAM sequence or specify it using standard nucleic acid alphabet characters. The user may also allow a mismatch between a sgRNA and its genomic target sequence in the first 8 nucleotides. A recent study found that such a mismatch does not affect sgRNA binding [24] and is relevant to this software because targeting several sequences with the same sgRNA is more feasible if such a mismatch is allowed. Sequence input to this software can be accomplished simply by pasting the sequences or contents of FASTA files into the text area of the website or uploading to the program. Alternatively, the user can provide sequence identifiers, their corresponding species and the type of identifiers among gene symbols, Ensembl Gene/Transcript IDs, or RefSeq IDs.

### Processing multiple sequences

Sequence alignment was performed using ClustalW2 software [25] after the input sequence files had been automatically prepared by Python scripts. The output files from sequence alignment were then processed using Biopython [26] to generate alignment objects suitable for finding common guide RNA sites in multiple sequences.

### External datasets

The dataset of zebrafish ohnologs for the Zv9 zebrafish reference genome was obtained from the authors of the latest zebrafish genome assembly (Sanger Zebrafish Genome Consortium) [27] upon request. The ohnologs were defined in this study as “runs of genes in the non-duplicated species that are found on two different chromosomes in the species that underwent a whole-genome duplication”. The total number of genes is 8083 and the number of “pairs” is 3440 (S1 Table). Some of these “pairs” are pairs between groups of ohnologs. When these group pairs were split into all possible unique pairs of single genes, the total number of unique ohnolog pairs became 6305 (S2 Table).

### Data analysis, visualization and graphics

Results from genome- or transcriptome-wide analysis were processed initially using custom-written Python scripts and then imported into R language environment (R Studio) for plotting. Input data, all intermediate files, python and R scripts are available from the GitHub repository https://github.com/SergeyPry/CRISPR_MultiTargeter/. Some files were too big to include but their construction was explained in the same repository. All of the figures were generated using GIMP and Inkscape software.

### Types of sgRNA design in CRISPR MultiTargeter and their applications

The main motivation for developing the CRISPR Multiargeter tool was to provide an effective computational method to identify common and unique targets for sgRNAs of the CRISPR/Cas system in several similar sequences. Having such a set of target sites would reduce the number of sgRNAs in experiments which aim at the simultaneous disruption of multiple similar genes. Alternatively, a set of unique sites for each of the similar sequences would allow a more fine-tuned targeting approach.

**Fig 1:**
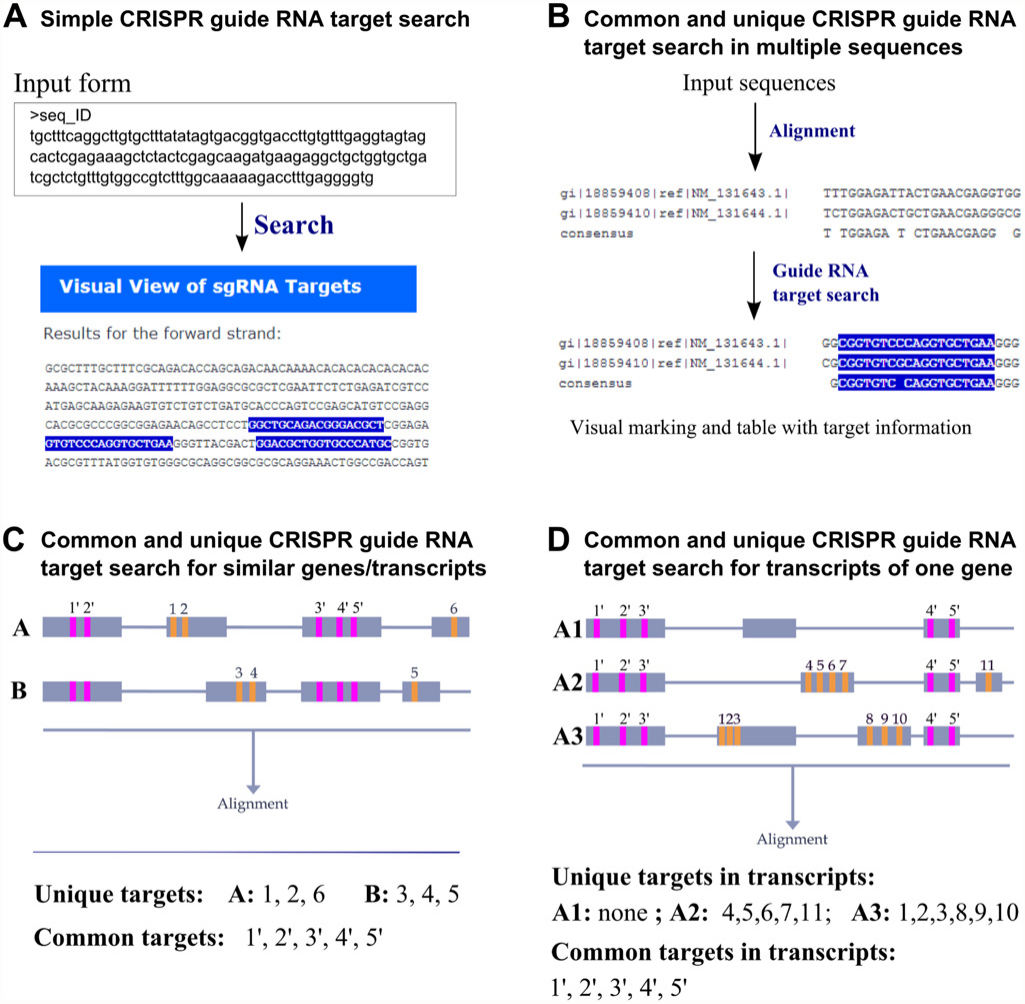
Workflows of guide RNA target search in CRISPR MultiTargeter. **A**. Simple CRISPR guide RNA search. A user enters a number of sequences or sequence identifiers and specifications for a target search. The program then runs these data, performs a regular expression match, stores the resulting coordinates and generates visual and table views of targets in each sequence. **B**. Common guide RNA target search in multiple sequences. Input sequences are used to generate a multiple sequence alignment. As in **(A)**, a regular expression with target specifications is run on the alignment consensus in both forward and reverse orientations. A successful match is defined as one having a maximum of one mismatch in the consensus sequence if the user allows mismatches. These matches are then highlighted in the multiple sequence alignment. In addition another algorithm is run on the input sequences to find unique target sites in each sequence (not shown). **C**. Common and unique guide RNA target search **in similar genes or transcripts**. In this workflow, gene or transcript sequences are retrieved from the database. Common targets are detected based on the multiple sequence alignment and unique target sites are found using an exhaustive string comparison algorithm (not shown). All targets sites are also checked to lie within a single exon to ensure successful targeting of the genomic sequence. In the illustration, locations of different target sites in genes A and B are shown together with the expected output of the program run. **D**. Common and unique guide RNA target search in transcripts of **a single gene**. Search for target sites is performed as described in **(C)**. In the illustration, input sequences are transcript isoforms A1, A2 and A3 of the gene A. The different types of target sites are shown as well as the expected program output. In **(C)** an **(D)**, common targets are indicated in pink and unique targets are in orange.

For ease of use, we generated four different workflows focusing on specific kinds of sgRNA target design. The simple CRISPR sgRNA search page allows the user to find target sites in one or more input sequences according to the defined sgRNA target site specificity (Fig. 1A). The regular expression target specificity implementation ensures that all possible targets can be found even if they overlap. Moreover, the program supports the design of sgRNAs for the normal single-cut double-stranded endonuclease activity and nearby pairs of sgRNAs for Cas9-nickase [28] applications. There are also multiple ways for the user to provide input sequences: one can input a DNA sequence without any identifier or as FASTA-formatted text, upload the same sequences in a file or provide sequence identifiers (for usage, see the next section), which will be used to retrieve the corresponding sequences from the website database. In addition, we wanted to provide a simple design experience for new users and provide them the opportunity to consider other types of sgRNA design available on the website. Although other CRISPR sgRNA design tools provide similar functionality, CRISPR MultiTargeter is not limited to the currently most used Type II CRISPR/Cas9 system but distinguishes itself by being able to accommodate new CRISPR/Cas system specificities such as NmCas9 [9] and StCas9 [10] (Table 1).

The next three workflows of CRISPR MultiTargeter work only with multiple sequences and share the main features of the algorithm, which generates a multiple sequence alignment from these sequences to find common targets and performs an exhaustive string comparison to identify unique target sites in each sequence. They differ in the mechanism of sequence input and their ability to check whether each identified target site is located within a particular exon. The first of these workflows focuses on multiple sequences provided in the FASTA format without any regard for the provenance of these sequences or their exon structure in the genome due to lack of such information (Fig. 1B). The main rationale for developing this workflow was to enable users to perform highly customized target searches using our tool in cases where those sequences have not been added to the main model system databases or when derived from the genomes of species not yet supported by the program. CRISPR MultiTargeter also features the same type of analysis as applied to similar genes or transcripts retrieved from the database by their sequence identifiers (Fig. 1C) or for DNA representations of different transcript isoforms of a single gene (Fig. 1D). In both of these latter two workflows, the species of origin of the sequences is known, as well as their exon structure. Gene sequences used in these workflows were built by merging all exons of each gene as described in the Database creation section.

### Input, algorithm, and output of CRISPR MultiTargeter

**Fig 2:**
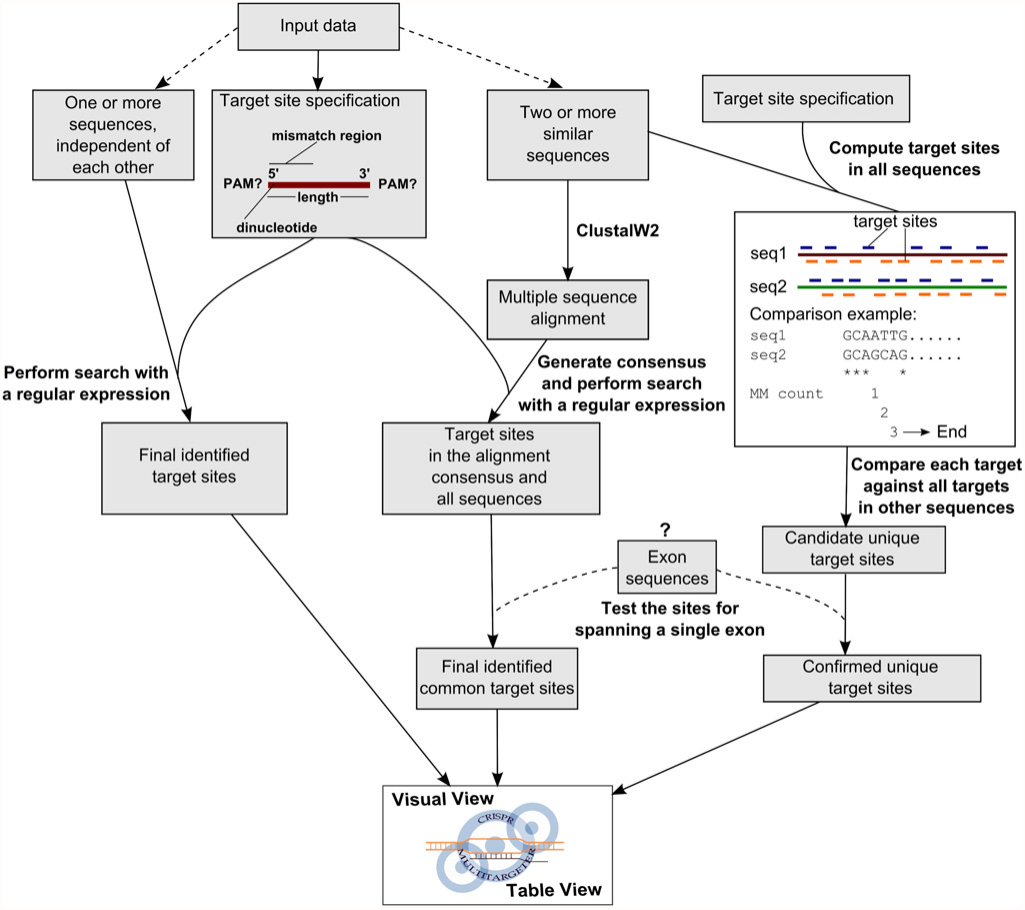
Search algorithm for sgRNA target sites in individual and multiple similar sequences. Input data for this algorithm consist of a sgRNA target site specification and sequence data. The dashed lines to the sequence boxes represent two possible branches of the algorithm: simple CRISPR sgRNA search and a search for common and unique target sites in multiple similar sequences. Target site specification is common to both branches of the algorithm and consists of a target site length, PAM sequence and its location as well as the sequence of the 5’-dinucleotide and the region where a single mismatch is allowed. The simple sgRNA search is achieved by running a regular expression (search pattern) for the target site specification on all input sequences in both orientations. The program can provide output for the sequence and location of identified target sites in visual and table formats. In the second branch of the algorithm, multiple similar sequences are first aligned using the ClustalW2 program. The resulting multiple sequence alignment is read by the program and the consensus sequence is computed. Running the target site specification expression on this consensus sequence results in the identification of candidate common target sites. If exon sequences are available for a particular sequence (indicated by “?” and dashed lines), each candidate target site in both common and unique sets is checked to ensure that this site lies completely within an exon sequence. Final identified common target sites are then displayed in visual and table formats. The search for unique target sites is accomplished by computing all possible target sites in both orientations in all sequences. Each target site is then compared to all identified target sites in these sequences. The speed of comparison depends on a mismatch count variable (MM count), which ensures that the comparison is stopped (“End”) as soon as there are more than 2 mismatches (identities are indicated by “*”). The target sites which pass this comparison test and the subsequent test for location within exon sequences are confirmed unique target sites. These unique target sites can then be output as before.

CRISPR MultiTargeter requires several parameters of CRISPR sgRNA targets and input sequences along with their associated parameters (Fig. 2). Due to the mechanism of the CRISPR/Cas system action, the main sgRNA characteristics are length of the target site and the PAM sequence appropriate for the particular type of CRISPR/Cas system. Recent experiments have explored the target site length and mismatch tolerance of the Type II CRISPR/Cas system [24,29,30]. The typical length of a target site in this system is 17–20 nucleotides, which results in N_17–20_NGG target site definition for the CRISPR/Cas9 system. Another implemented parameter is whether the 5’-most dinucleotide is completely unconstrained (NN) or must conform to another pattern generated by sgRNA synthesis by T7 polymerase (GG) or from the U6 promoter (GN). Overall, the current implementation of target site searches is based on the assumption that the only sequence constraints of CRISPR/Cas target sites are the 5’-most dinucleotide and PAM sequence. One of the main proposed uses of CRISPR MultiTargeter is to target multiple sequences, which can be more easily achieved by allowing mismatches in the first 8 nucleotides not known to significantly affect sgRNA binding [24]. This option may not generalize well to other systems, so the user may choose not to allow any mismatches. The mismatch option may be extended in subsequent versions of the program if such mismatch data become available for other systems. As discussed previously, this implementation is therefore sufficient for the available CRISPR/Cas system tools and can be expected to adapt well to novel systems because they share target site determination by sgRNAs. Input sequences are processed by the algorithm according to whether the simple or alignment-based sgRNA target search is being performed.

**Fig 3:**
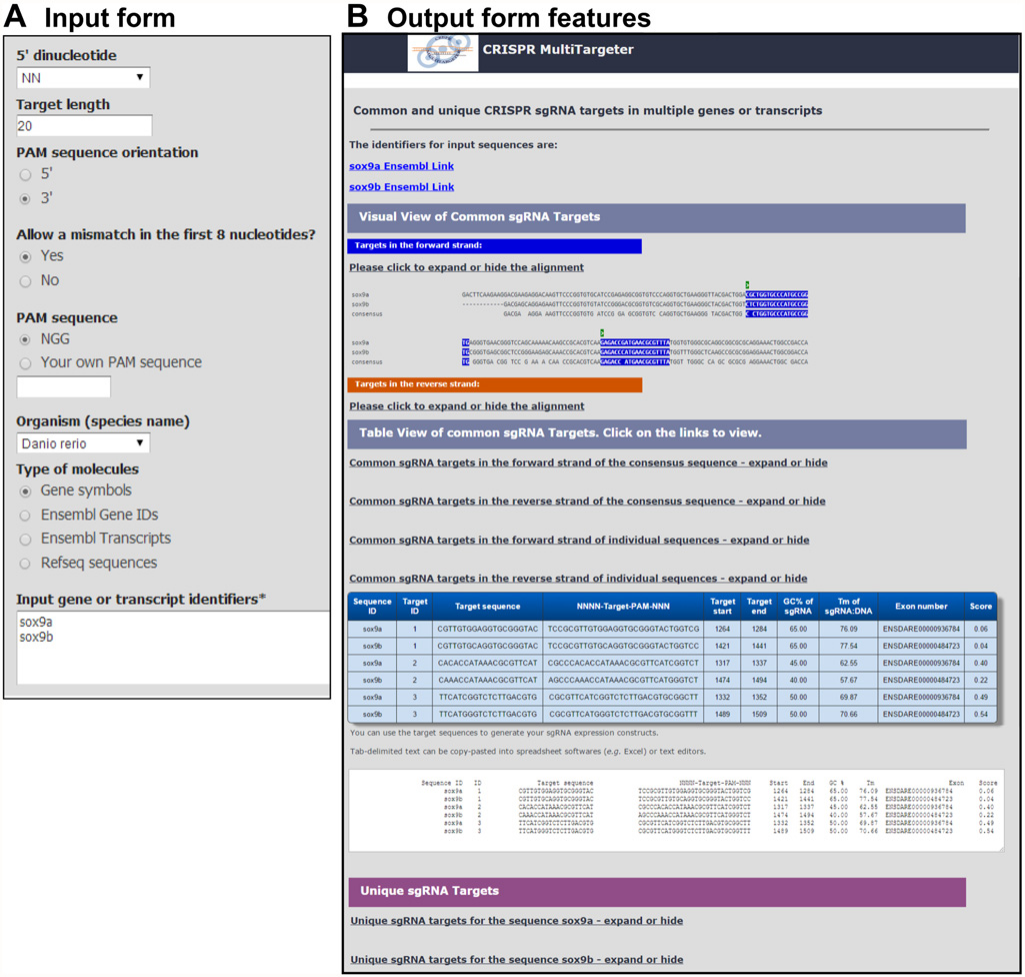
Examples of input and output pages of CRISPR MultiTargeter. **A**. The input form for the multiple genes or transcripts input consists of the parameters for the sgRNA target site specification and the identifiers input area. **B**. The output page consists of the overall header indicating the type of design performed followed by the list of input identifiers which the user provided with the links to Ensembl gene pages where available. This example is from the multiple genes/transcripts workflow performed on *sox9a* and *sox9b* zebrafish genes. The main part of the output is focused on common sgRNA target sites and is organized in Visual and Table Views. The user can see the details of these views by clicking on the “expand or hide” links. Visual View consists of links to alignment with the target sites highlighted and markers for the start sites of target sites. Table View contains HTML tables with the relevant information on sgRNA target sites such as their ID numbers, sequences, start, end as well as computed sequence features such as GC % and predicted annealing temperature (Tm) of sgRNA:DNA interaction, exon numbers and predicted scores. There is also a “Unique sgRNA targets” part of the page which is organized similarly.

In the simple search algorithm, one or more DNA sequences are taken as input and searches are performed independently of each other (Fig. 2). Once all the target sites in all sequences are found, the program provides visual and table views of the target sites in expandable and collapsible (implemented in JavaScript) links to alignments with labeled target sites and tables with target site sequence data (Fig. 3). Tables are provided in HTML and as text for pasting into spreadsheets or text editors.

In contrast, the main algorithm of the program focusing on finding common and unique target sites processes the input sequences based on the assumption that they are similar. First, a FASTA file suitable for ClustalW2 is written temporarily to a new folder. CRISPR MultiTargeter then runs ClustalW2, which computes an alignment of input sequences and writes the resulting output files to the same folder. The output alignment is then read using Biopython and a consensus sequence for the whole alignment is computed. The representation of gaps (“-”) and mismatches (“X”) allows the program to easily perform a regular expression search on the consensus sequence according to the criteria defined above. This search is then performed using standard Python code on both the consensus sequence and its reverse complement. To ensure the correct output, the algorithm keeps track of the target site coordinates in the consensus sequence and in each individual sequence in both orientations. Next, unique sgRNA target sites are computed by a completely different algorithm (Fig. 2). First, all possible target sites are computed for each sequence in both orientations. Since target sites without a PAM sequence are mostly non-functional for cleavage by Cas proteins, it is possible to limit testing to uniqueness of target sites only fornormal PAM-containing sites. Each of the identified target sites is then compared with all other target sites in all sequences. The comparison is performed from the 5’-end and if the number of differences between them is more than two, the comparison stops and the program moves on to the next target site (Fig. 2). Both common and unique sgRNA target sites in sequences with known exon structures are also filtered by checking that they are located completely within a single exon to ensure that their sequences are not a result of RNA splicing of two different exons and therefore will not occur in the genome (Fig. 2).

### Calculating characteristics of sgRNAs

Each identified sgRNA target site is characterized by its GC percentage, which affects the melting temperature (Tm) of the resulting RNA: DNA duplex, which may affect activity of an sgRNA but is not directly proportional. Although it is difficult to predict the exact Tm of this duplex inside CRISPR/Cas protein complexes, it is possible to predict it based on *in vitro* biophysical experiments and computational models derived from them. We implemented the thermodynamical nearest-neighbor Tm prediction originally provided by Sugimoto et al.,1995 [31] and based on a previous implementation in MELTING [32]. The python code was derived from Bio.SeqUtils.MeltingTemp Biopython package and the results were verified against the results of MELTING 4.2 (http://mobyle.pasteur.fr/cgi-bin/portal.py?#forms::melting). Another parameter implemented in CRISPR MultiTargeter is the score from 0 to 1 indicating the predicted activity of sgRNAs and derived from a logistic regression model described by Doench and colleagues [19]. Briefly, the scoring function the sgRNA target sequence, 4 nucleotides (nt) to the 5’, PAM sequence and 3 nucleotides to the 3’ of PAM. The current requirement for the scoring function that the sgRNAs are of type II and are 20 nt long.

### Off-target testing of predicted sgRNAs

Since the task of off-target analysis involves complex computations involving large amounts of genomic data and this analysis is not the main focus of the paper, we decided to add explanations and links to the tool output, which will enable the user to analyse the identified target sites for potential off-targets using other tools. We selected Cas-OFFinder (http://www.rgenome.net/cas-offinder/) [22] and GT-Scan (http://gt-scan.braembl.org.au/gt-scan/) [23] as the tools for the down-stream off-target analysis. In both cases, the user has to copy text input area output containing sgRNA target site sequences and their characteristics into a spreadsheet program and then select the sgRNA target site sequences for analysis. There are four parameters to select for an off-target analysis: the type of PAM sequence, sequence length, target genome and the maximum mismatch number of potential off-target sites. Cas-OFFinder allows 4 different PAM types and the associated sequence lengths are fixed in this program, whereas GT-Scan is very flexible in this regard and allows the user to specify any type of PAM sequence and choose the sequence length. Cas-OFFinder and GT-Scan also have good coverage of target genomes with 23 and 28 genomes available, respectively, and most model species are present among these genomes. Finally, both programs allow the user to specify the maximum number of mismatches between a sgRNA target site and potential off-target sites in the genome.

## Results and Discussion

### Unique sgRNA target site identification in zebrafish transcript isoforms using CRISPR MultiTargeter

**Fig 4:**
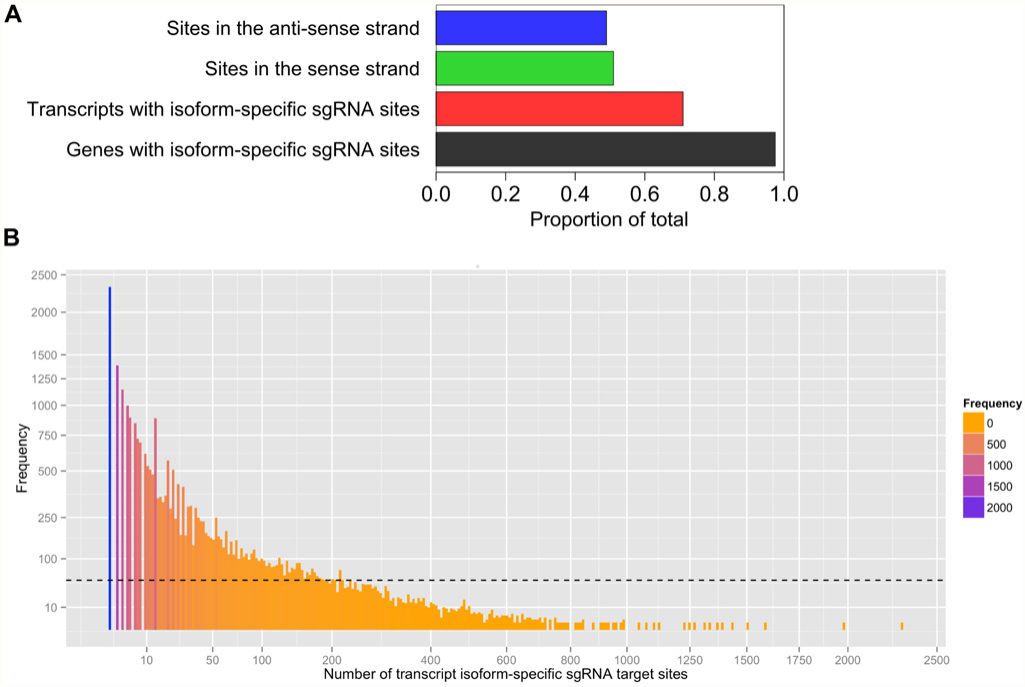
Unique transcript isoform-specific sgRNA target sites for Type II CRISPR sgRNAs in zebrafish genes. **A**. Proportions of genes with identified transcript isoform-specific sgRNA sites, transcripts with isoform-specific sgRNA sites and proportions of these sites in the sense and anti-sense orientation. sgRNA sites are 20 bp long with the NN 5’-dinucleotide and NGG PAM sequence. **B**. Distribution of total target site numbers for transcript isoforms. The mean number of sgRNA target sites (48.7) is indicated by a dashed line over the histogram. The graph axes are scaled using the square root function. The histogram bars are colored according to the frequency scale as shown.

To demonstrate the applicability of CRISPR MultiTargeter for determining unique transcript isoform-specific sgRNA target sites, we decided to focus on a set of zebrafish genes with multiple alternative transcripts. Unique sgRNA target sites are highly relevant for the mutational analysis of specific transcript isoforms. Indeed, targeting specific transcript isoforms in the mouse using CRISPR/Cas technology has been proposed in a recent review on subtle targeted mutations, which are becoming increasingly important for understanding gene function [33]. Until recently, such targeting experiments were very challenging in the mouse and nearly impossible in species not amenable to genome engineering by homologous recombination, like the zebrafish. Application of CRISPR MultiTargeter for designing transcript isoform-specific sgRNAs will greatly simplify the design of such experiments. The genome-wide uniqueness of these isoform-specific sgRNAs can be examined using either Cas-OFFinder [22] or GT-Scan web tool [23] as described in Methods. If defined mutations need to be introduced, co-injections of sgRNA and Cas9 with relevant double- or single-stranded DNA molecules can be performed. In this genome-wide analysis in zebrafish, we used a standalone Python script of the CRISPR MultiTargeter transcript workflow with the default settings (5’-dinucleotide—NN; length—20; PAM sequence—NGG) to search for transcript-isoform specific (unique) sgRNA target sites in all zebrafish genes with two or more isoforms according to the Ensembl database. Transcript isoforms are present in about 40% of all zebrafish genes based on our analysis and a total number of genes in the Zv9 zebrafish reference genome [27]. We analysed these 12,383 genes using our workflow for transcripts of a single gene to identify and quantify unique transcript isoform-specific sgRNA target sites (Fig. 4). Nearly all of these genes (97.5%) had at least one transcript with unique sgRNA target sites, which can be expected as the identification of unique sites will only require a sufficient length of alternative exonic sequence in alternative transcripts (Fig. 4A). Likewise, 71% of all alternative transcripts analysed in this program contained unique isoform-specific sgRNA sites (Fig. 4A). This percentage can be explained by the fact that many alternative exons are present in multiple known transcript isoforms and thus would not qualify for uniqueness in the set of transcripts of a single gene. sgRNA sites found in this analysis are almost equally distributed among the sense and anti-sense orientations (51% and 49%, respectively) (Fig. 4A). The distribution of the transcript isoform-specific sgRNA numbers in individual transcripts is fairly broad but biased toward lower frequencies with a mean of 48.7 (Fig. 4B). Such a broad distribution of target numbers reflects the variability in the length of transcript isoform-specific sequence regions. Overall, this analysis of zebrafish transcript isoforms shows that sgRNA target sites can be identified in individual transcript isoforms by CRISPR MultiTargeter and thus facilitate isoform-specific targeting experiments. The resulting more advanced mutational analysis will likely improve our understanding of the role of the products of alternative transcripts in the cell.

### Application of CRISPR MultiTargeter to duplicated genes in zebrafish to identify and quantify common sgRNA target sites

**Fig 5:**
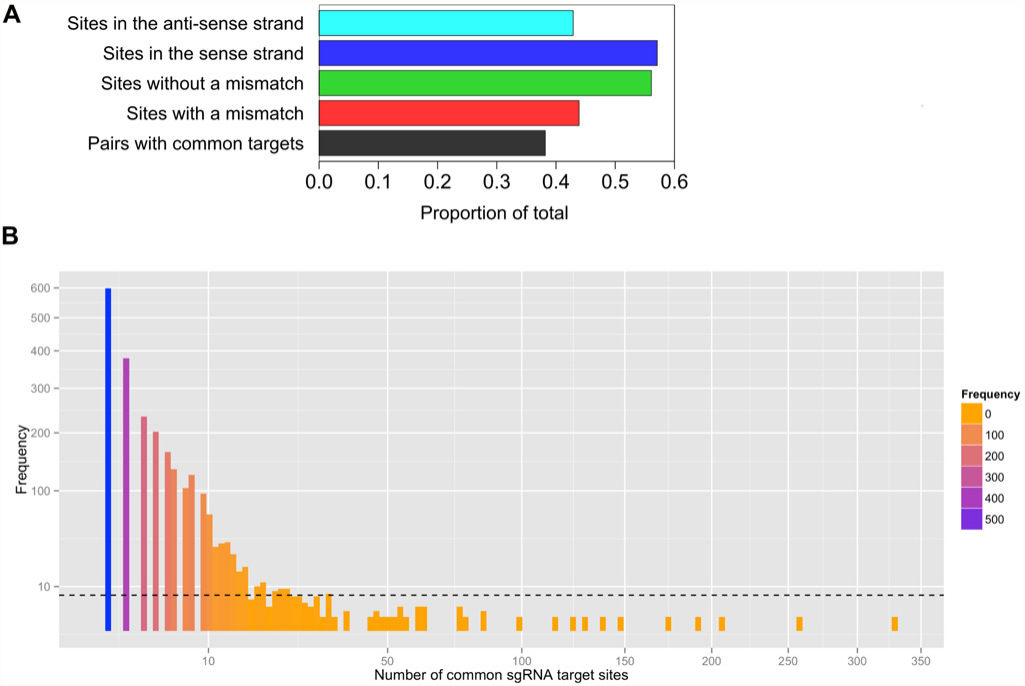
Common target sites for Type II CRISPR sgRNAs in zebrafish ohnologs. **A**. Proportions of gene pairs with identified common sgRNA target sites, target sites with single mismatches and without mismatches, as well as in the sense and anti-sense strands. sgRNA sites are 20 bp long with the NN 5’-dinucleotide and NGG PAM sequence. **B**. Distribution of total common target site numbers for different gene pairs. The mean number of target sites (6.48) is indicated by a dashed line over the histogram. The graph axes are scaled using the square root function. The histogram bars are colored according to the frequency scale as shown.

To test CRISPR MultiTargeter at identifying common targets in similar genes we chose a set of zebrafish ohnologs, i.e. paralogous genes originating from genome duplications and named in honour of Susumu Ohno [34]. Understanding ohnologs and orthologous relationships of genes in different species is important for a correct orthology-based nomenclature of genes, evolutionary studies, and for an easier dissection of gene functions. In the zebrafish genome, it is a frequent occurrence that generation of loss-of-function models for certain genes is complicated by the presence of very similar duplicated genes thus requiring the investigators to target at least two genes to achieve a significant phenotype. Evidence from whole-genome sequencing such as the order of genes and synteny of regions between species suggests that the lineage leading to the origin of zebrafish has undergone two rounds of genome duplication during the origin of vertebrates and an additional one during teleost evolution (reviewed in [35,36]). After genome duplications, many of the duplicate genes are differentially lost in diverging lineages, resulting in apparently novel genes, while other genes acquire novel functions and/or expression patterns, which can contribute to evolutionary innovation [37]. Thus, gene duplications and subsequent gene losses can promote lineage divergence and a greater genetic and morphological complexity. Evolutionary considerations aside, practical work on targeting two or more ohnologs requires identification of the most complete set of ohnolog pairs or groups. Currently, the most robust methods are based on identification of large-scale regions of conserved synteny between species [38,39]. The set of ohnologs identified in the latest zebrafish reference genome paper [27] was chosen to test the applicability of CRISPR MultiTargeter to design sgRNAs targeting similar genes. These authors used the double-conserved synteny zebrafish to human comparison method to identify 3440 pairs of ohnologs and a total of 8083 ohnologs, which represent 26% of all zebrafish genes. Some of these ohnolog pairs are pairs between groups of ohnologs. To simplify our testing application, paired groups were split into all possible pairs, which resulted in 6305 pairs of genes. A simplified version of CRISPR MultiTargeter with default parameters was used to identify common targets for each pair of genes. Common target sites were identified for 2412 pairs of ohnologs (38.2%, Fig. 5A), which indicates significant applicability of CRISPR MultiTargeter for such genes. Recent findings that 17-nt sgRNA target sites are more specific and no less potent [30] may further raise the percentage of targetable ohnologs. The proportion of target sites with single mismatches (43.9%, Fig. 5A) was smaller than that of fully conserved target sites (56.1%, Fig. 5A), suggesting that common sgRNA target sites can more likely be found in regions highly conserved among similar sequences. Interestingly, common sgRNA target sites were also more prevalent in the sense orientation of multiple sequence alignments (57.1%, Fig. 5A) than in the anti-sense orientation (42.9%, Fig. 5A), which can be explained by a biased distribution of “GG” sequence in different DNA strands of conserved alignment regions. The distribution of common target site counts for different pairs of ohnologs is strongly skewed toward small numbers of target sites, with some genes showing higher numbers of common target sites (Fig. 5B). The results of this computational analysis indicate a broad applicability of CRISPR MultiTargeter to target both duplicated genes simultaneously with one sgRNA. Such an application can be useful to simplify the targeting of one or more pairs of duplicated genes. As the originally demonstrated multiplex genome editing with CRISPR/Cas9 system [40] is now applied to different species and purposes [41,42], it will be a frequent occurrence that some of the genes have duplicates, which the user may want to target. Alternatively, if a gene has known ohnologs, CRISPR MultiTargeter can identify target sites for this gene not present in the other ohnologs without performing whole-genome searches. Finally, to address off-target activity concerns, the user can apply one of the available off-target prediction softwares [21,22].

### Comparison of CRISPR MultiTargeter to other sgRNA design tools

To highlight why the current software can be useful for successful sgRNA design and provide a comparative analysis of many other software tools, we compiled several important features of sgRNA design tools (Table 1). It is possible that some tools are not included, but this overview is meant to be representative and not all-inclusive. Most of the current software tools support either the currently dominant Type II CRISPR Cas9 system from *Streptococcus pyogenes* or multiple Type II systems from other bacterial species. In contrast, CRISPR MultiTargeter supports all of these and other CRISPR/Cas system if the user can specify the position (5’ or 3’) relative to the target site and sequence of PAM using the standard nucleic acid alphabet. Although this feature is not of immediate importance, other CRISPR/Cas systems are likely to be adopted for experimental use and thus will require new software features similar to what we implemented. Like other software tools, CRISPR MultiTargeter runs as a web-based tool, accepts both sequence input and several different types of identifiers, supports both wild-type and Cas9 nickase design types, contains a database of sequences from multiple model systems and agriculturally important species and like 4 other tools (Optimized CRISPR Design, sgRNAcas9, sgRNA Designer, CRISPRseek) supports analyses in a batch mode. Unlike other tools, we did not implement an off-target analysis of individual sgRNAs designed using our tools because it was not the main focus of the software, but rather provide detailed instructions to the user both on the input and output pages on how to search for off-targets using recently developed and dedicated web tools Cas-OFFinder [22] and GT-Scan [23], two programs that integrate well with CRISPR Multitargeter. Interestingly, the sgRNA Designer tool [19] authors also did not implement off-target analysis due to potential irrelevance of the off-target cleavage to the experimental purpose at hand and the computational expense involved. The main purpose of sgRNA Designer, however, was to implement a model for sgRNA scoring based on high-throughput experiments in cell lines, which analysed the effects of 1841 type II 20-nt sgRNAs on 9 genes. This model represents logistic regression of nucleotide preferences at different positions as well as some global features such as GC content. We implemented this model in an unmodified form in CRISPR MultiTargeter with the expectation that it may help users select more potent sgRNAs. However, we found that scores for a number of effective sgRNAs were between 0.1 and 0.2 (unpublished observations). Therefore, some guidance is provided on the website so the user can interpret these scores. Scoring systems for sgRNAs are still in their infancy and additional studies in several model systems are necessary to verify the predictions of sgRNA effectiveness made by this model with a need to develop additional models.

Although the features discussed above are important, the main rationale for CRISPR MultiTargeter was to look at several similar sequences and identify the sgRNA target sites shared by these sequences or unique to one of them. Among the currently available tools, the only one allowing somewhat similar functionality is CRISPRseek [18] developed as a Bioconductor package and capable of identifying sgRNA target sites having different cleavage efficiencies between two very similar sequences such as alleles of a gene. Such an application was not implemented in CRISPR MultiTargeter since it is aimed at analyzing a set of more dissimilar sequences. The two tools may therefore have complementary uses and the approach similar to the one in CRISPRseek can also be implemented in CRISPR MultiTargeter as an additional workflow. Regardless of the similarities and differences between these two tools, we believe that availability of such tools as CRISPR MultiTargeter and CRISPRseek will promote the design of CRISPR/Cas targeting by focusing not only on an isolated gene or site but on a broader context of different alleles, similar genes and transcript isoforms.

## Conclusions

We developed the CRISPR MultiTargeter web tool to support mutational targeting and genome engineering using the recently developed CRISPR/Cas system. This software has two distinguishing features in its application: First, easy definition of novel sgRNA target site specification and, second, given a set of similar sequences, an opportunity to identify target sites common to all of these sequences and also those unique to each particular sequence in a set. Similar to other sgRNA design software, we also provide the design of sgRNAs for mutational targeting by wild-type Cas9 and nickase Cas9 mutants, as well as several options for target site definition, sequence input and a convenient output format. We also implemented a recently developed quality scoring algorithm [19]. CRISPR MultiTargeter can currently be applied to the genomes of nine animal,three plant species as well as to Refseq nucleotide sequences from any species and additional species databases can be easily added. There are three different algorithms for searching sgRNA target sites. The user can search for sgRNA sites by matching individual sequences with a sgRNA target site definition. For common sgRNA targets, multiple sequence alignment followed by target site matching of the consensus sequence is easily accomplished. By contrast, identification of unique target sites in each sequence requires using a string comparison algorithm between all possible target sites in different sequences. The program was computationally tested by finding transcript isoform-specific sgRNAs in all alternative transcripts in the zebrafish, which revealed the broad applicability of the tool for this task and significant potential for the transcript isoform-specific mutational analysis in many species. A second trial of the program focused on duplicated genes in the zebrafish and resulted in common target site identification is almost 40% of gene pairs. Such common target sites can be used for simultaneous gene pair inactivation in multi-gene inactivation experiments. In the zebrafish, the prevalence of duplicated genes is about 26% thus making such an application quite relevant. In summary, we propose that CRISPR MultiTargeter will complement existing tools for CRISPR sgRNA design and facilitate new types of genetic analysis.

### Availability and requirements

This software is freely available for use at the www.multicrispr.net. The source code for the website scripts, standalone scripts and instructions for constructing databases are available at https://github.com/SergeyPry/CRISPR_MultiTargeter.

## Supporting Information

S1 Table Ohnolog pairings from the zebrafish reference genome paper. (XLSX)

S2 Table All possible ohnolog pairs in the zebrafish genome used for common target site analysis. (XLSX)
