# Correction: CRISPR MultiTargeter: A Web Tool to Find Common and Unique CRISPR Single Guide RNA Targets in a Set of Similar Sequences

**DOI:** 10.1371/journal.pone.0138634

**Published:** 2015-09-14

**Authors:** Sergey V. Prykhozhij, Vinothkumar Rajan, Daniel Gaston, Jason N. Berman

The following information is missing from the Funding section: Vinothkumar Rajan is funded through Cancer Research Trainee Program of the Beatrice Hunter Cancer Institute, Nova Scotia in partnership with the Canadian Imperial bank of Commerce and New Brunswick Health Research Foundation. The funders had no role in study design, data collection and analysis, decision to publish, or preparation of the manuscript.
